# Dysphagia With Unusual Etiology: A Case Report

**DOI:** 10.7759/cureus.40000

**Published:** 2023-06-05

**Authors:** Nehemias A Guevara, Jorge Sanchez, Garry Francis-Morel, Ming Yu, Ricardo Velasquez

**Affiliations:** 1 Internal Medicine, St. Barnabas Hospital Health System, Bronx, USA; 2 Critical Care Medicine, St. Barnabas Hospital Health System, Bronx, USA

**Keywords:** dysphagia lusoria, hemiazygos vein, vascular dysphagia, rare cause of dysphagia, progressive dysphagia

## Abstract

Here, we present a case of dysphagia with a very unusual etiology. Dysphagia is a symptom of concern and can occur secondary to multiple etiologies. Therefore, prompt and appropriate evaluation is necessary, as treatment varies depending on the underlying cause. Our patient was a 73-year-old female admitted for dysphagia associated with recent significant weight loss and a history of long-term smoking. A CT scan of her neck revealed a mass that was compressing the esophagus, but the etiology of the mass was unexpected. This case highlights the importance of considering rare causes of dysphagia and underscores the need for physicians to be aware of them.

## Introduction

Dysphagia is defined as impairment or difficulty in swallowing, hindering the passage of food from the mouth to the stomach [1]. The underlying causes of dysphagia are broad and can be categorized as oropharyngeal or esophageal dysphagia. Esophageal dysphagia can result from mechanical obstruction, inflammatory diseases, or motility disorders [2]. On the other hand, oropharyngeal dysphagia symptoms arise from the dysfunctional transfer of the food bolus in the pharynx to the upper esophageal sphincter into the esophagus and most commonly observed in the elderly population, with an incidence of up to 16% after the 50 years old [3]. Nevertheless, external vascular compression of the esophagus is a rare etiology of dysphagia and a critical differential diagnosis. Most of the time, due to aberrant vascular abnormality, usually arterial, vascular compression is even less likely to be seen. We present a rare case of dysphagia in an orderly patient who was initially suspected of having a tumor but was ultimately diagnosed with a different condition.

## Case presentation

A 73-year-old female with a medical history of asthma, osteoporosis, prediabetes, and a 2-pack-year history of smoking presented to the Emergency Department with a one-month history of progressive dysphagia to solids and liquids. Additionally, she reported experiencing right-sided neck pain, unintentional weight loss of 10-20 pounds within the past month, sputum production, mild chest pain, night sweats, and Globus sensation. Her family history was unremarkable. Physical examination was unremarkable except for right posterior cervical lymphadenopathy, and initial laboratory workup showed a normal blood count and basic metabolic panel. 

**Figure 1:**
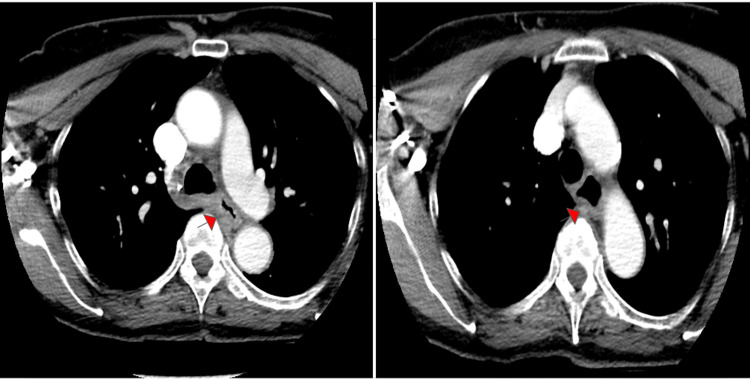
CT neck images Mild mural thickening of the upper thoracic esophagus (red arrows), with compression and collapse of the lumen at the level of the carina by a 3.5 x 2.7 cm mass, which was suspicious for a neoplasm, with differentials including but not limited to esophageal leiomyoma, gastrointestinal stromal tumors, and esophageal carcinoma

Computed tomography (CT) of the neck revealed abnormalities associated with malignancies, such as esophageal leiomyoma, gastrointestinal stromal tumors, and esophageal carcinoma (Figure 1).

**Figure 2:**
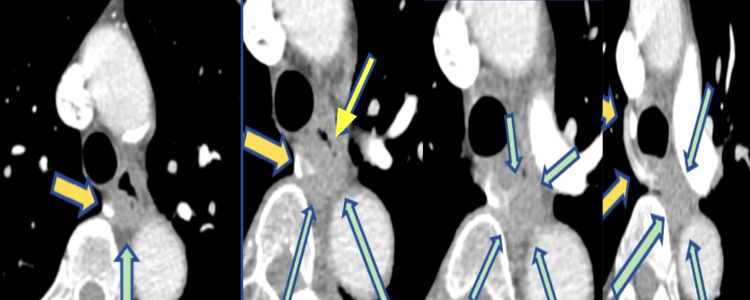
CT chest images Images show a 1.36 x 1.77 x 1.96 cm soft tissue mass along the esophagus's posterior wall (blue-green arrows) at the distal trachea and T5-T7 vertebrae level as a right apical pleural-based thick-walled soft tissue density. Yellow arrows show a hemizygos vein with a thickening of the wall.

Chest CT showed a soft tissue mass on the posterior wall of the esophagus and a right apical pleural-based thick-walled soft tissue density (Figure 2). CT scans of the abdomen and pelvis were unremarkable.

**Figure 3:**
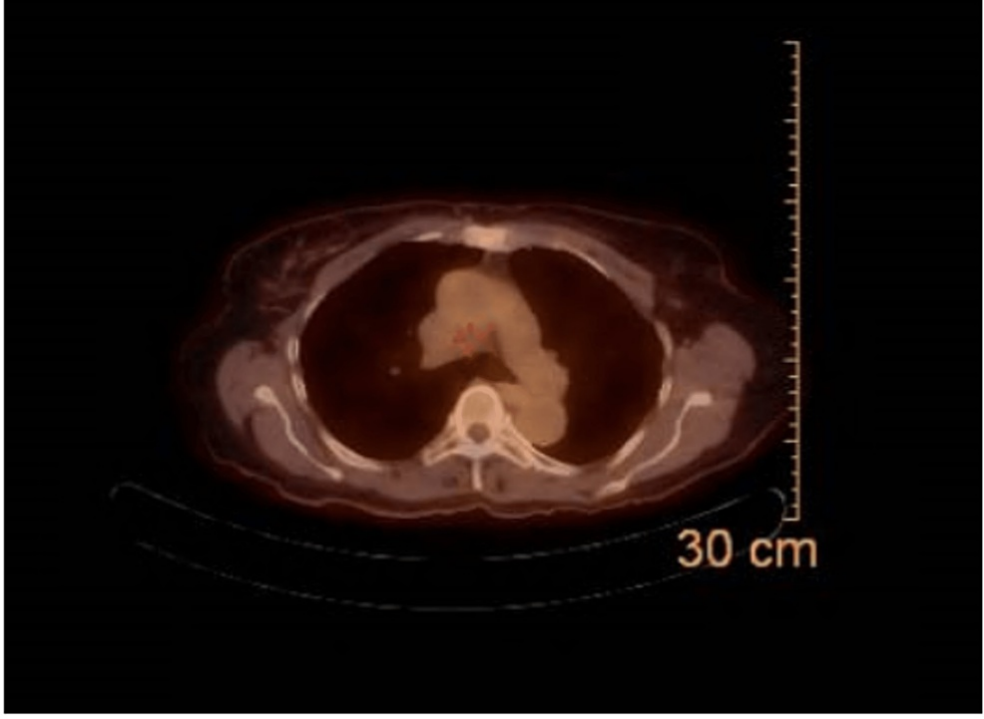
PET-scan, did not show any FDG PET: positron emission tomography; FDG: fluorodeoxyglucose

The patient underwent upper endoscopy, which revealed a non-bleeding gastric ulcer with a clean base (Forrest Class III) and a single stomach papule, but no other abnormalities were found. Pathology results indicated acute inflammation of the squamous epithelium in the lower esophagus, suggestive of reflux esophagitis, with no signs of malignancy. Based on these findings, interventional radiology recommended a positron emission tomography (PET)-Scan, which showed a mass adjacent to the mid-thoracic esophagus. The PET-Scan indicated no significant increase in metabolic activity, and the mass appeared to be a prominent hemizygos vein (Figure 3). The patient was referred for cardiovascular surgery, which she currently follows, but no surgical plan has been made.

## Discussion

Dysphagia is a sensation of difficulty swallowing associated with solid foods or liquids. Solid food-associated dysphagia is usually due to mechanical obstruction, while motility disorders typically cause dysphagia associated with both solid and liquid food. However, severe dysphagia can occur even with liquids [4,5]. A population-based survey conducted by Adkins et al. found that about 16% of the US population experienced dysphagia. The most commonly reported causes were gastroesophageal reflux disease (GERD), eosinophilic esophagitis, and esophageal strictures, accounting for 30.9%, 8%, and 4.5%, respectively. Other causes, such as diffuse esophageal spasm, esophageal infection, achalasia, and scleroderma, were also reported [6].

In a study by Mitra et al., 35% of dysphagia cases were caused by malignancy, with squamous cell carcinoma of the esophagus being the most common (71.7%), followed by adenocarcinoma of the esophagus (25.7%). GERD was the most common benign cause of dysphagia in both studies. All patients with malignancy in the study by Mitra et al. were habitual tobacco users, which was also a suspected cause of dysphagia in the present case [7].

Vascular etiology is rare but is a critical cause of dysphagia that can result from external compression. Arterial abnormalities or aneurysms are the most common causes of vascular etiology. Dysphagia lusoria is a type of dysphagia resulting from extrinsic esophageal compression caused by an aberrant right subclavian artery (ARSA). It is characterized by an aberrant course behind the esophagus of the right subclavian artery and is an embryological defect of the aortic arch vasculature. The right subclavian artery is most commonly involved (0.5%-2.0%), and the left subclavian artery is rarely involved when there is a right-sided aortic arch (0.05-0.1%) [8]. Dysphagia lusoria typically presents as chronic dysphagia with no alarming qualities [9,10]. Surgical decompression is the treatment of choice for vascular repair [11]. In the present case, the patient was referred for cardiothoracic surgery but declined surgical intervention and continues to be followed up. Due to their structure, minimal to no muscle, and easy compressibility, Venous abnormalities are a rare cause of dysphagia within vascular etiologies. Hence, when a patient presents with dysphagia, it is usually at the bottom of the differential list [12].

This report discusses a case of dysphagia secondary to mass effect in a patient, not due to a neoplasm but caused by an enlarged hemiazygos vein. The hemiazygos vein is part of the azygos system that drains venous blood from the posterior aspect of the axial skeleton of the trunk and has three major components: the azygos vein proper that drains the venous blood of the right side of the posterior trunk; its counterpart to the hemiazygos vein, which drains the left lower thorax; and the accessory hemiazygos vein which drains the left upper thorax, all located in the posterior mediastinum. The hemiazygos vein ends around T8 by joining the azygos vein [13,14].

The posterior mediastinum has several structures that, once altered, can cause dysphagia; however, enlargement of the azygos vein, let alone the hemiazygos vein, is one of the rarest causes described [15]. This enlargement can occur either due to direct obstruction or collateralization, leading to an indentation or pressure in the esophagus and resulting in the previously explained symptoms [16]. The patient, in this case, exhibited dysphagia to solids and liquids, night sweats, and weight loss, along with being an active everyday smoker, which raised the possibility of an esophageal malignancy as the top differential diagnosis.

Odedra et al. reported a case with symptoms of chest pain and dysphagia symptoms, and a chest radiograph showing a shadow in the paratracheal area suggesting malignancy. However, a subsequent biopsy showed no evidence of malignancy or granulomas, and a thorough review of diagnostic images revealed that an engorged azygos vein was partially obstructing the esophagus [17]. This was the only reported case similar to ours, albeit with the azygos vein rather than the hemiazygos vein causing the external esophageal compression. Therefore, our case is the first reported instance of the hemiazygos vein causing external esophageal compression mimicking a malignancy.

The above-mentioned case report emphasized the importance of re-evaluating patients with symptoms that do not correspond to diagnostic findings. This can prevent patients from undergoing unnecessary procedures, laboratory tests, and potentially fatal treatments. Our case is a prime example of how a seemingly straightforward clinical scenario can lead to an unexpected and rare outcome.

## Conclusions

This case highlights the importance of considering uncommon causes of dysphagia. Our patient had red-flag symptoms that are not typically associated with vascular causes. Despite this, dysphagia can have many underlying causes, with benign causes being the most common. Nonetheless, conducting a proper diagnostic approach for patients with risk factors is crucial.
